# Non-coplanar volumetric-modulated arc therapy (VMAT) for craniopharyngiomas reduces radiation doses to the bilateral hippocampus: a planning study comparing dynamic conformal arc therapy, coplanar VMAT, and non-coplanar VMAT

**DOI:** 10.1186/s13014-016-0659-x

**Published:** 2016-06-23

**Authors:** Megumi Uto, Takashi Mizowaki, Kengo Ogura, Masahiro Hiraoka

**Affiliations:** Department of Radiation Oncology and Image-applied Therapy, Kyoto University Graduate School of Medicine, 54 Shogoin Kawahara-cho, Sakyo-ku, Kyoto, 606-8507 Japan

**Keywords:** Hippocampus, Craniopharyngioma, Dosimetric comparison, Dynamic conformal arc therapy, Coplanar VMAT, Noncoplanar VMAT

## Abstract

**Background:**

Recent studies suggest that radiation-induced injuries to the hippocampus play important roles in compromising neurocognitive functioning for patients with brain tumors and it could be important to spare the hippocampus using modern planning methods for patients with craniopharyngiomas. As bilateral hippocampus are located on the same level as the planning target volume (PTV) in patients with craniopharyngioma, it seems possible to reduce doses to hippocampus using non-coplanar beams. While the use of non-coplanar beams in volumetric-modulated arc therapy (VMAT) of malignant intracranial tumors has recently been reported, no dosimetric comparison has yet been made between VMAT using non-coplanar arcs (ncVMAT) and VMAT employing only coplanar arcs (coVMAT) among patients with craniopharyngiomas. We performed a planning study comparing dose distributions to the PTV, hippocampus, and other organs at risk (OAR) of dynamic conformal arc therapy (DCAT), coVMAT, and ncVMAT.

**Methods:**

DCAT, coVMAT, and ncVMAT plans were created for 10 patients with craniopharyngiomas. The prescription dose was 52.2 Gy in 29 fractions, and 99 % of each PTV was covered by 90 % of the prescribed dose. The maximum dose was held below 107 % of the prescribed dose. CoVMAT and ncVMAT plans were formulated to satisfy the following criteria: the doses to the hippocampus were minimized, and the doses to the OAR were similar to or lower than those of DCAT.

**Results:**

The mean equivalent doses in 2-Gy fractions to 40 % of the volumes of the bilateral hippocampus [EQD_2_(40%_hippos_)] were 15.4/10.8/6.5 Gy for DCAT/coVMAT/ncVMAT, respectively. The EQD_2_(40%_hippos_) for ncVMAT were <7.3 Gy, which is the threshold predicting cognitive impairment, as defined by Gondi et al.. The mean doses to normal brain tissue and the conformity indices were similar for the three plans, and the homogeneity indices were significantly better for coVMAT and ncVMAT compared with DCAT.

**Conclusions:**

NcVMAT is more appropriate than DCAT and coVMAT for patients with craniopharyngiomas. NcVMAT significantly reduces radiation doses to the bilateral hippocampus (to 50 % that of the DCAT) without increasing the doses to normal brain tissue and other OAR.

**Electronic supplementary material:**

The online version of this article (doi:10.1186/s13014-016-0659-x) contains supplementary material, which is available to authorized users.

## Background

Patients with craniopharyngiomas exhibit a bimodal age distribution, and peaks are evident at ages 5–14 and 50–74 years [1]. Aggressive surgery is often associated with increased frequencies of neurological, visual, cognitive, and neuroendocrinological side effects compared with limited surgery [2–4]. Therefore, limited surgery followed by radiotherapy (RT) is often used to manage craniopharyngiomas. This multidisciplinary approach (limited surgery and RT) affords a 10-year overall survival (OS) rate of 70–83 % and a 10-year progression-free survival (PFS) rate of 60–69 % [5–7].

As patients with craniopharyngiomas make good prognoses [5–7] and as pediatric patients seem to be more sensitive to radiation than adults, irradiation of normal tissue should be minimized. Cognitive decline is a recognized late effect of cranial irradiation, and it is suspected that radiation-induced injuries to the hippocampus are major contributors to neurocognitive deficits in patients with brain tumors [8–11]. The hippocampus is located close to the planning target volumes (PTVs) for craniopharyngiomas, and it could be important to spare the hippocampus using modern planning methods.

In terms of radiation techniques, 3D conformal external beam radiotherapy (3D-CRT) delivered using dynamic conformal arc therapy (DCAT), intensity-modulated radiotherapy (IMRT), and volumetric-modulated arc therapy (VMAT) are clinically employed to treat craniopharyngiomas [12]. In VMAT, both the shape of the radiation beam and the beam modulation can be changed while the gantry is rotating. VMAT reduces treatment delivery time and monitor units, and target coverage is equal to or better than that of IMRT [13]. VMAT can spare the hippocampus using inverse planning method but it remains unclear whether such sparing might increase the doses to other organs at risk (OAR). In addition, the use of non-coplanar beams in IMRT and VMAT for malignant intracranial tumors has recently been reported [14–16]. However, to the best of our knowledge, no dosimetric comparison has yet been made between VMAT using non-coplanar arcs (ncVMAT) and VMAT employing only coplanar arcs (coVMAT) for craniopharyngiomas with focus particularly on the hippocampal doses.

Therefore, we performed a planning study comparing the dose distributions to the PTVs and OAR of DCAT, coVMAT, and ncVMAT and identified the technique that maximally reduced doses to the hippocampus of patients with craniopharyngiomas.

## Methods

This study followed all dictates of the Helsinki declaration and our Institutional Ethical Review Board approved the research (approval number E–1802).

### Patient population

Ten patients with histologically confirmed craniopharyngiomas who were treated at our institution from November 2009 to November 2014 were included.

### Target and OAR delineation

Contouring and treatment planning were performed using previously acquired computed tomography (CT) images and the Eclipse version 11.0.47 (Varian Medical Systems, Palo Alto, CA, USA). CT images 1.25-mm thick were acquired by a Light Speed RT scanner (GE Healthcare, Milwaukee, WI, USA). Patients were immobilized in thermoplastic masks with bite blocks. Pre- and post-operative magnetic resonance imaging (MRI) scans were fused with the planning CT images.

As cystic lesions change in size during radiation therapy [17] and as residual tumors were possibly present, each clinical target volume (CTV) was defined to include any residual gross tumor and 5-mm thicknesses of any normal brain tissue attached to each tumor on preoperative MRI imaging. Then, the PTV was defined as the CTV plus 2-mm margin to allow for setup errors and patient motion. The lenses, eyes, optic nerves, chiasm, brainstem, hippocampus, and normal brain tissue were contoured as OAR. The hippocampus was delineated as described by Marsh et al. [18]. Couch structures were contoured and included in calculations.

### Treatment planning

DCAT, coVMAT, and ncVMAT plans were created for each of 10 cases. Six-megavolt photon beams delivered by a Varian CL21iX linear accelerator through a Millennium 120-leaf multileaf collimator (Varian Medical Systems) were used in all plans. The Acuros XB dose Acurous calculation algorithm was employed; the calculation grid size was 2.5 mm x 2.5 mm. The dose prescribed for the PTV was 52.2 Gy in 29 fractions, and all plans were normalized to ensure that V90 = 99 % (thus, 99 % of the PTV was covered by 90 % of the prescribed dose). The maximum doses to the brainstem, optic nerves/chiasm, and lens were set at less than 54, 55, and 10 Gy, respectively.

### DCAT plans

**Fig. 1:**
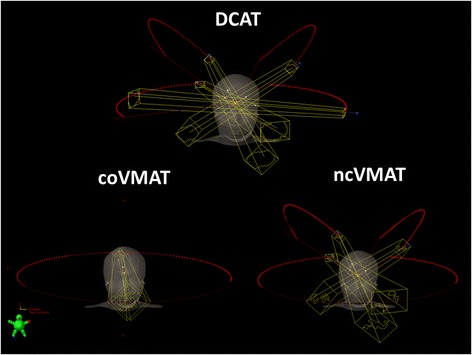
Beam arrangement in a representative case; DCAT, coVMAT, and ncVMAT. DCAT = dynamic conformal arc therapy, coVMAT = coplanar volumetric-modulated arc therapy, ncVMAT = noncoplanar volumetric-modulated arc therapy

Each DCAT plan consisted of two coplanar and two non-coplanar arcs. We ensured that no beam irradiated the eyes. Two non-coplanar arcs were placed at couch angles of 45° and 315° (Fig. 1). All collimeter angles were set to 0°. The maximum dose (Dmax) was held below 107 % of the prescribed dose.

### coVMAT plans

Each coVMAT plan was created using the RapidArc system (Varian Medical Systems) and coplanar double arcs. One arc rotated clockwise from 181° to 179°, and the collimator angle was 45°. The other arc rotated counterclockwise from 179° to 181°, and the collimator angle was 315°. For both arcs, the couch positions were set to 0°, and avoidance sectors were placed to ensure that the eyes were not irradiated.

Dmax was set to be below 107 % (as in DCAT planning). Optimization was performed until the final result met the following criteria: the doses to the eyes, lenses, optic nerves, chiasm, and brainstem were similar to or lower than those of DCAT and the dose to the hippocampus was maximally reduced.

### ncVMAT plans

Each ncVMAT plan consisted of one coplanar arc and two non-coplanar arcs. We employed the RapidArc System. The coplanar arc was rotated clockwise with avoidances sectors not to irradiate the eyes. The couch positions and arc rotations of the two noncoplanar arcs were the same as those of DCAT. All collimater angles were set to 0°. Optimization was performed to ensure that the coVMAT criteria (see above) were met. As with the DCAT and coVMAT plans, Dmax was set to be below 107 %.

### Evaluation of treatment plans

The DCAT, coVMAT, and ncVMAT plans were compared in terms of target homogeneity, target conformity, and the volumes of OAR irradiated. The homogeneity index (HI) was defined as (D2% − D98%)/D50%, where D2%, D98%, and D50% were the doses covering 2 %, 98 %, and 50 % of the PTV, respectively. Two conformity indices (CIs) were calculated. One was the RTOG-CI, defined as V_(90)_/V_PTV_, where V_(90)_ was the volume enclosed by 90 % of the prescription isodose surface, and V_PTV_ was the PTV. The other index was Ian Paddick’s conformity index (IP-CI) described by Paddick et al.[19] and that was defined as V_PTV(90)_^2^/(V_(90)_ x V_PTV_). V_PTV(90)_ is the volume of the PTV receiving 90 % of the prescribed dose.

We calculated the equivalent doses in 2-Gy fractions to 40 % of the bilateral hippocampus [EQD_2_(40%_hippos_)] as well as D40%_hippo_, which were the doses covering 40 % of the volume of the bilateral hippocampus. The EQD_2_(40%_hippo_) index developed by Gondi et al. can be used to predict long-term neurocognitive functioning [9] and was derived from the D40%_hippo_, assuming an α/β ratio of 2.

As the PTV varied individually, we also calculated normalized D40%_hippo_ values (NV_D40%_hippos_). That of DCAT was set to unity. The NV_D40%_hippo_ of coVMAT and ncVMAT were defined as D40%_hippo_(coVMAT)/D40%_hippo_(DCAT) and D40%_hippo_(ncVMAT)/D40%_hippo_(DCAT), respectively. We evaluated the doses to PTV and OAR using D2% and D98%.

### Statistical analysis

All statistical analyses were performed with the aid of EZR (Saitama Medical Center, Jichi Medical University; http://www.jichi.ac.jp/saitama-sct/SaitamaHP.files/manual.html  [20]), which is a graphical user interface for R (the R Foundation for Statistical Computing, Vienna, Austria, version 3.0.2). More precisely, EZR is a modified version of R commander version 1.24, facilitating biostatistical evaluations.

Data from the three planning techniques (DCAT, coVMAT, and ncVMAT) were compared using two-way analysis of variance (ANOVA) across the entire cohort, followed by Bonferroni post hoc testing. A P-value <0.05 was considered to indicate statistical significance.

## Results

### Target coverage

The median PTV was 20.0 cm^3^ (range, 7.23–51.08 cm^3^). Table 1 summarizes the PTV index values. The HIs of coVMAT and ncVMAT were significantly better than those of DCAT (p < 0.05). We found no significant difference among the three techniques in the RTOG-CI, IP-CI, or the mean PTV dose.

**Table 1:** Summary of indices about the PTV

Index	DCAT	coVMAT	ncVMAT	*P*-value (ANOVA)	*P*-value (DCAT vs. coVMAT)	*P*-value (DCAT vs. ncVMAT)	*P*-value (coVMAT vs. ncVMAT)
	(Mean ± SD)						
HI	0.114 ± 0.010	0.103 ± 0.008	0.099 ± 0.005	<0.05	<0.05	<0.05	0.676
RTOG-CI	1.758 ± 0.672	1.378 ± 0.138	1.429 ± 0.175	0.112			
IP-CI	0.615 ± 0.164	0.717 ± 0.069	0.694 ± 0.078	0.123			
D2% (Gy)	54.0 ± 0.6	53.1 ± 0.6	52.9 ± 0.5	<0.05	<0.05	<0.05	1
D98% (Gy)	48.0 ± 0.2	47.8 ± 0.2	47.8 ± 0.2	0.101			
Dmean (Gy)	52.3 ± 0.4	51.7 ± 1.3	51.4 ± 0.5	0.077			

If a significant difference was evident when data from the entire cohort were compared via two-way analysis of variance (ANOVA), the Bonferroni post hoc test was performed to compare pairs of modalities

*PTV* planning target volume, *DCAT* dynamic conformal arc therapy, *coVMAT* coplanar volumetric-modulated arc therapy, *ncVMAT* non-coplanar volumetric-modulated arc therapy

*HI* homogeneity index, *CI* conformity index, *RTOG-CI* CI as defined by the Radiation Therapy Oncology Group (RTOG), *IP-CI* CI as defined by Paddick et al. [19], *D2%* dose to 2 % of the volume, *D98%* dose to 98 % of the volume, *Dmean* mean dose

### Normal tissue doses

**Fig. 2:**
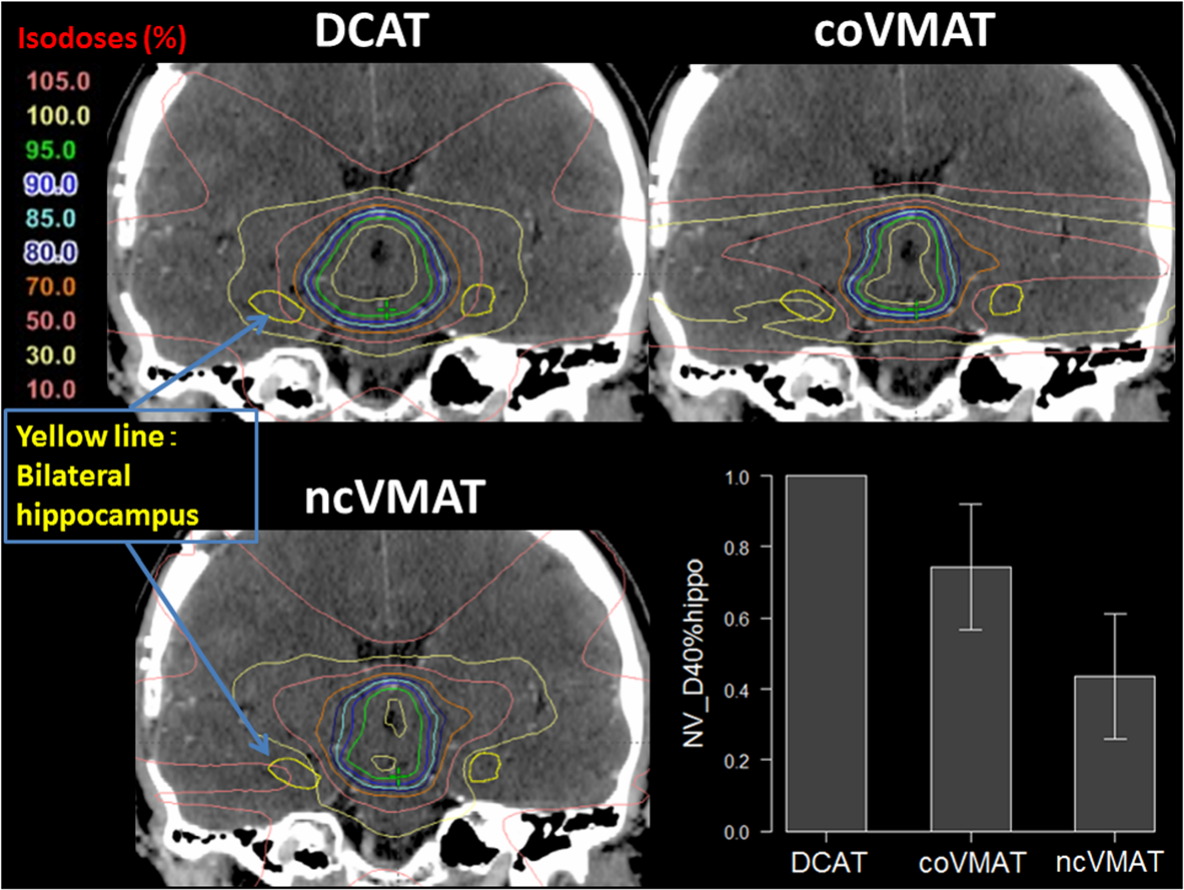
Coronal plains of dose distributions in a representative case and a comparison of the normalized doses covering 40 % of the volume of the bilateral hippocampus using DCAT, coVMAT, and ncVMAT. The *yellow line* shows the contour of the bilateral hippocampus. The normalized value of D40%_hippo_ indicates that the dose covering 40 % of the volume of the bilateral hippocampus was significantly reduced in the following order: ncVMAT, coVMAT, and DCAT (ncVMAT 0.4, coVMAT 0.7, and DCAT 1). DCAT = dynamic conformal arc therapy, coVMAT = coplanar volumetric-modulated arc therapy, ncVMAT = non-coplanar volumetric-modulated arc therapy, NV_D40%_hippo_ = normalized value of D40%_hippo_

Table 2 summarizes doses to the hippocampus and normal brain tissue. Mean doses to the bilateral hippocampus, D40%_hippo_ and EQD_2_(40%_hippo_) emerged in the following order: ncVMAT, coVMAT, and DCAT. The mean EQD_2_(40%_hippo_) were 6.5, 10.8, and 15.4 Gy for ncVMAT, coVMAT, and DCAT, respectively. The mean EQD_2_(40%_hippo_) for ncVMAT were <7.3 Gy, which is the threshold dose to indicate cognitive impairment, as defined by Gondi et al. [9]. The NV_D40%_hippo_ index tended to emerge in a similar manner and with statistical significance (ncVMAT: 0.4, coVMAT: 0.7, and DCAT: 1). The mean doses to normal brain tissue were similar in the three plans. Single coronal slices showing the dose distributions of each plan in a representative case are presented in Fig. 2.

**Table 2:** Summary of doses delivered to the hippocampus and normal brain, in Gy

Structure/index	DCAT	coVMAT	ncVMAT	*P*-value (ANOVA)	*P*-value (DCAT vs. coVMAT)	*P*-value (DCAT vs. ncVMAT)	*P*-value (coVMAT vs. ncVMAT)
	(Mean ± SD)						
Bilateral Hippo							
D2%	36.5 ± 11.2	29.5 ± 12.6	23.1 ± 15.5	0.095			
Mean dose	20.7 ± 7.6	15.4 ± 8.1	10.7 ± 8.2	<0.05	0.515	<0.05	0.530
D40%_hippo_	21.7 ± 8.4	16.2 ± 7.6	10.3 ± 7.9	<0.05	0.406	<0.05	0.326
EQD_2_(40%_hippo_)	15.5 ± 7.9	10.8 ± 6.3	6.5 ± 6.0	<0.05	0.41	<0.05	0.51
NV_D40%_hippo_	1	0.7 ± 0.2	0.4 ± 0.2	<0.05	<0.05	<0.05	<0.05
Normal brain							
Mean dose	6.8 ± 1.7	6.2 ± 1.5	6.8 ± 1.4	0.563			

If a significant difference was evident when data from the entire cohort were compared via two-way analysis of variance (ANOVA), the Bonferroni post hoc test was performed to compare pairs of modalities

*DCAT* dynamic conformal arc therapy, *coVMAT* coplanar volumetric-modulated arc therapy, *ncVMAT* non-coplanar volumetric-modulated arc therapy, *SD* standard deviation, *Hippo* hippocampus

*D2%* dose to 2 % of the volume, *D40%*_*hippo*_ dose to 40 % of the volume of the bilateral hippocampus

*EQD*_*2*_ *(40%*_*hippo*_ *)* equivalent dose in 2-Gy fractions (assuming α/β = 2) to 40 % of volume of the bilateral hippocampus

*NV_D40%*_*hippo*_ normalized value of D40%_hippo_ (the DCAT value was set to unity). The normalized values for coVMAT and ncVMAT were calculated as D40%_hippo_(coVMAT)/D40%_hippo_(DCAT) and D40%_hippo_(ncVMAT)/D40%_hippo_(DCAT), respectively

The D2% to both lenses were lower in the ncVMAT than in the other plans. There was no significant difference among the plans in the D2% to the optic nerves.

Doses to OAR, excluding the hippocampus and normal brain tissue, are summarized in Table 3.

**Table 3:** Summary of OAR doses (hippocampus and normal brain tissue excluded)

Structure/index	DCAT	coVMAT	ncVMAT	*P*-value (ANOVA)	*P*-value (DCAT vs. coVMAT)	*P*-value (DCAT vs. ncVMAT)	*P*-value (coVMAT vs. ncVMAT)
	(Mean ± SD)						
Lt. optic nerve							
D2%	38.4 ± 11.9	37.2 ± 11.2	36.2 ± 12.8	0.915			
Rt. optic nerve							
D2%	43.6 ± 9.5	40.5 ± 10.9	41.4 ± 10.5	0.789			
Chiasm							
D2%	53.3 ± 0.8	52.2 ± 0.9	52.4 ± 0.7	<0.05	<0.05	<0.05	1
Lt. lens							
D2%	3.2 ± 0.7	2.2 ± 0.7	1.9 ± 0.4	<0.05	<0.05	<0.05	0.854
Rt. lens							
D2%	3.2 ± 0.7	2.3 ± 0.7	1.8 ± 0.5	<0.05	<0.05	<0.05	0.224

If a significant difference was evident when data from the entire cohort were compared via two-way analysis of variance (ANOVA), the Bonferroni post hoc test was performed to compare pairs of modalities

*D2%* dose to 2 % of the volume

*SD* standard deviation, *DCAT* dynamic conformal arc therapy, *coVMAT* coplanar volumetric-modulated arc therapy, *ncVMAT* noncoplanar volumetric-modulated arc therapy, *Lt.* left, *Rt.* right

## Discussion

We compared the dose distributions of DCAT, coVMAT, and ncVMAT plans for craniopharyngioma patients. NcVMAT delivered a significantly lower dose to the bilateral hippocampus than did the other two techniques; the mean EQD_2_(40%_hippo_) of ncVMAT was <7.3 Gy, which is the threshold predicting cognitive decline according to Gondi et al. [9]. NcVMAT achieved a better HI without increasing OAR doses. We thus found that ncVMAT was the best method to spare the bilateral hippocampus compared with DCAT and coVMAT.

Neurocognitive toxicity after cranial irradiation is often seen in patients who received whole brain radiotherapy (WBRT) to treat metastatic brain tumors [11, 21]. Many reports have explored the mechanism underpinning this phenomenon, and the dose to the temporal lobes seemed to predict the decline [22, 23]. Among temporal lobes, the hippocampus is considered important in terms of learning ability and memory functioning after irradiation [8, 10, 21, 24, 25]. The hippocampus are located in the median-temporal lobes, and Gondi et al. suggested that the hippocampal doses predicted impairments in the neurocognitive functioning of patients with benign or low-grade brain tumors [9]. A dose–response relationship seemed to be evident between the EQD_2_(40%_hippo_) and the impairment in Wechsler Memory Scale-III Word List. Word recall was delayed 18 months after irradiation; the EQD_2_(40%_hippo_) threshold predicting cognitive impairment was 7.3 Gy.

Neurocognitive decline is often seen in patients with craniopharyngiomas, especially pediatric patients. Greenfield et al. found that 33 % of pediatric patients who underwent surgery and IMRT exhibited neurocognitive and behavioral deficits at the last follow up and reported that a larger PTV was significantly associated with development of neurocognitive problems [5]. The greater the PTV, the closer the bilateral hippocampus was to that PTV; thus, the doses to the hippocampus may increase linearly with a rise in PTV. Therefore, hippocampal irradiation may contribute to cognitive decline in patients with craniopharyngiomas as well as other intracranial tumors, and it would be better to minimize doses to the hippocampus.

3D-CRT and VMAT are used to treat craniopharyngiomas in clinical practice, but the optimal method remains unknown. About 75 % of craniopharyngiomas develop in the suprasellar region, close to the bilateral hippocampus, and it is difficult to set the EQD_2_(40%_hippo_) below 7.3 Gy when DCAT is planned [1]. VMAT can spare the hippocampus using an inverse planning method. In 2009, Wiggenraad et al. compared DCAT and non-coplanar IMRT (ncIMRT) in patients with various intracranial tumors [26]. When target conformity, homogeneity, and doses to the optic nerves and chiasm were all considered, the cited authors concluded that DCAT was equal to or better than ncIMRT in six of seven patients with skull-base meningiomas. The locations of such meningiomas are similar to those of craniopharyngiomas, and we found that ncVMAT afforded a better HI than and a similar CI to DCAT while providing equal or lower doses to other OAR. The reason for the between-study difference is not clear, but it is possible that the use of VMAT rather than IMRT, and the method of optimization, are important to achieve better dose distribution.

We found that the HI of ncVMAT was significantly better than that of DCAT. Although statistical significance was lacking, the HI of ncVMAT was slightly better than that of coVMAT. It is well known that craniopharyngiomas undergo transient enlargement during radiation therapy [17]. The optic nerves and the chiasm are close to the PTV, and the HI is thus important in craniopharyngioma patients. If the target homogeneity is to be prioritized, ncVMAT may be more appropriate than DCAT or coVMAT.

Noncoplanar arcs are usually used in DCAT to improve PTV conformity and homogeneity [12]. Recently, non-coplanar beams in IMRT and VMAT have been found to be useful for treating intracranial malignant tumors [14–16]. A study on the utility of non-coplanar beams in IMRT and VMAT for the treatment fronto-temporal high-grade gliomas found that such beams reduced the doses to contralateral OAR, including the anterior/temporal lobes and optic structures [16]. The structures thus spared were usually coplanar and close to the PTVs of patients with fronto-temporal high-grade gliomas. The hippocampus is also located on the same level as the PTV in patients with craniopharyngiomas, and it appears that the use of ncVMAT compared with coVMAT may reduce the doses to coplanar structures. To the best of our knowledge, this is the first report to declare the utility of ncVMAT in terms of dose reductions to the hippocampus of patients with craniopharyngiomas.

One limitation of this study must be mentioned. This planning study compared dose distributions during DCAT, coVMAT, and ncVMAT, but it is unclear whether reducing the dose to the hippocampus actually assists in the preservation of cognitive functioning in clinical situations. Indeed, other factors contribute to the development of neurocognitive disorders in patients with irradiated craniopharyngiomas. These include the tumor per se, surgery, doses to the temporal lobes, shunt placement, presence of an Ommaya reservoir, diabetes insipidus, and low pre-irradiation growth hormone levels [5, 23, 27, 28]. Prospective clinical trials on craniopharyngioma patients are required to explore whether the dosimetric advantage described herein affords real clinical benefits.

## Conclusions

NcVMAT is more appropriate than DCAT and coVMAT for patients with craniopharyngiomas. NcVMAT significantly reduces the dose to the bilateral hippocampus (to 50 % that of the dose for DCAT) without increasing the doses to normal brain tissue and other OAR. NcVMAT also improves target homogeneity.

### Availability of data and materials

The dataset supporting the conclusions of this article is included within the supplementary material (Additional file 1).

### Ethical approval and consent to participate

This study followed all dictates of the Helsinki Declaration and the ethical review board of Kyoto University Hospital and Faculty of Medicine approved the research (approval number E–1802). Written consent to participate was obtained from the patient.

### Consent to publish

Written consent was obtained from the patient for publication of this report and any accompanying images.

## Abbreviations

3D-CRT, 3D conformal external beam radiotherapy; CI, conformity index; coVMAT, volumetric-modulated arc therapy employing only coplanar arcs; CT, computed tomography; CTV, clinical target volume; D2%, dose to 2 % of the volume; D40%_hippo_, doses covering 40 % of the volume of the bilateral hippocampus; Dmax, maximum dose; EQD_2_(40%_hippos_), equivalent doses in 2-Gy fractions to 40 % of the volumes of the bilateral hippocampus; HI, homogeneity index; IMRT, intensity-modulated radiotherapy; MRI, magnetic resonance imaging; ncIMRT, non-coplanar intensity-modulated radiotherapy; ncVMAT, volumetric-modulated arc therapy using non-coplanar arcs; NV_D40%_hippos_, normalized values of doses covering 40 % of the volume of the bilateral hippocampus; OAR, organs at risk; OS, overall survival; PFS, progression-free survival; PTV, planning target volume; RT, radiotherapy; V_(90)_, volume enclosed by 90 % of the prescription isodose surface ; VMAT, volumetric-modulated arc therapy; V_PTV(90)_, volume of the planning target volume receiving 90 % of the prescribed dose; V_PTV_, planning target volume; WBRT, whole brain radiotherapy.

## Additional file

Additional file 1: The dataset supporting our findings. (XLSX 26 kb)
